# Effectiveness of Anticoagulants in Reducing Stroke Risk Among Patients With Atrial Fibrillation

**DOI:** 10.7759/cureus.59298

**Published:** 2024-04-29

**Authors:** Sai Namrata Dasari, Sai T Gadde, Pravallika Myneni, Monicaa Bodduluri, Sindhu Chowdary Valiveti

**Affiliations:** 1 Internal Medicine, All India Institute of Medical Sciences, Mangalagiri, Vijayawada, IND; 2 General Medicine, All India Institute of Medical Sciences, Mangalagiri, Vijayawada, IND; 3 General Medicine, Katuri Medical College and Hospital, Vijayawada, IND; 4 Internal Medicine, Katuri Medical College and Hospital, Vijayawada, IND; 5 General Medicine, Sri Venkateswara Institute of Medical Sciences (SVIMS) Sri Padmavathi Medical College for Women (SPMCW), Tirupati, IND

**Keywords:** cardiac arrhythmia, neurological consequences, quality of life, oral anticoagulants, thromboembolic events, hemodynamic effects, cardiovascular disease, anticoagulant therapy, stroke prevention, atrial fibrillation

## Abstract

Atrial fibrillation (AF) is a type of cardiac arrhythmia causing shortness of breath, lightheadedness, and palpitations. It may go unrecognized and asymptomatic among many patients. AF is not a potentially fatal arrhythmia; its hemodynamic, structural, and hemocoagulative effects have a significant impact on the standard of life, which can lead to various complications such as stroke. A stroke caused by AF leads to additional burdens on both patients and the global economy. Patients with AF can prevent strokes with oral anticoagulants; however, ensuring diligent adherence to medication is crucial for maximizing treatment efficacy. Since they have a lighter treatment load than warfarin, non-vitamin K antagonist oral anticoagulants (NOACs) are also recommended with better hope for medication adherence. Various anticoagulants such as warfarin and ximelagatran, among many more, are prescribed to patients who have the potential to reduce the incidence of stroke as well as alleviate their likelihood of developing other thromboembolic events that can decrease their quality of life. Economic and psychological burdens associated with diminished functionality can be prevented by anticoagulant therapy among AF patients, therefore reducing their economic and social burden. This is due to the negative association between stroke among AF patients and anticoagulation consumption.

## Introduction and background

In the past few decades, atrial fibrillation (AF) has grown in importance as a matter concerning public health and a significant driver of healthcare spending in Western nations. Although AF is not a potentially fatal arrhythmia, its hemodynamic, structural, and hemocoagulative effects significantly impact the standard of life [1]. Recent guidelines, such as those outlined in the Journal of the American College of Cardiology, emphasize the importance of optimal management strategies for AF to mitigate its associated risks, particularly the increased risk of stroke [2]. These guidelines offer updated recommendations on the use of anticoagulants in AF patients, considering factors such as efficacy, safety, and patient adherence to treatment. People over the age of 70 years are more likely to experience AF, which is usually linked to hypertension, coronary artery heart disease, and cardiac failure. Recent studies have also highlighted the concerning relationship between AF and dementia, with approximately one in three AF patients suffering from dementia, often going unrecognized until late [3,4]. This underlines the complexity of managing AF and its associated comorbidities. Moreover, the evolving landscape of AF research has emphasized the need for comprehensive approaches to treatment and prevention, considering the interplay between AF and conditions such as coronary artery disease [5].

It has been reported that AF serves as an indicator of an elevated risk of stroke, with aging and associated circulatory irregularities cited as underlying reasons for the higher incidence of stroke among individuals with this condition. It is the second most vital potential causative agent for stroke and elevates the likelihood of suffering from stroke up to 5% [6-8]. The risk assessment for stroke in patients with AF often relies on tools such as the CHA_2_DS_2_-VASc score [9]. This scoring system takes into account various clinical factors, including congestive heart failure, hypertension, age, diabetes mellitus, prior stroke or transient ischemic attack, vascular disease, and sex category, to stratify patients into different risk categories for stroke.

According to a majority of significant research on the epidemiology of AF that was conducted in industrialized nations and reported approximately the final decades of the 20th century and the early years of the 21st century, the mean incidence of AF in the entire population was around 0.5% and 1% [10]. However, throughout the last 10 years, it was widely accepted that the prevalence of AF had to be significantly larger than what was indicated by the number of hospitalizations, emergency room visits, and cost of outpatient care for AF [11]. According to the most recent research, which ranged from 1.9% in Italy, Iceland, and England to 2.3% in Germany and 2.9% in Sweden, the frequency of AF in the overall adult population of Europe is over two times higher than what was previously recorded just a decade ago [12].

The number of Americans with AF, which is expected to reach 10 million by 2050, is a significant public health concern [13]. Congestive heart failure, stroke, and early mortality are all risks associated with it. With the rising use of antiarrhythmic medications, warfarin, and device treatments such as AF ablation during the past 20 years, AF therapy has changed [14]. According to certain research, the mortality risk for AF patients may have decreased over time, which raises the possibility that it may have altered for AF patients [15]. It has been reported that increased incidents of incidence of a hospital diagnosis of AF had doubled, which altered the 10-year mortality and morbidity rate. It had been reported to be 20% in men and 18% in women [15,16].

It was discovered that there is a $10,355 cost difference between individuals with AF and those without AF in terms of per capita medical costs. Individuals with AF who are 65 years of age or older also had greater overall costs. According to the report, untreated nonvalvular AF costs the US economy an additional $3.1 billion annually [17]. AF-caused stroke leads to additional economic as well as healthcare burdens on both patients and the global economy. The prevention of stroke via medication can not only alleviate the healthcare burden but also improve the overall survival rate of AF patients [18].

Stroke treatment and prevention have undergone significant strides in the last 10 years. Anticoagulation therapy is one of the most effective treatments for preventing strokes in people with AF [19]. AF patients also experience more severe strokes with much worse prognoses than individuals with sinus rhythm [20]. Up to 71% of people will die or have a serious neurological impairment, and over 10% will have another stroke each year [21]. Prevention is essential to lowering mortality and disability since stroke is frequently the first sign of an embolism [22]. Patients with AF can prevent strokes with oral anticoagulants, but careful drug compliance is essential to getting the most out of treatment [23]. Since they have a lighter treatment load than warfarin, non-vitamin K antagonist oral anticoagulants (NOACs) provide hope for improving adherence [24]. Warfarin is safe to be utilized in the medical management of AF and is beneficial in lowering the incidence of stroke [25]. It has been demonstrated to be economical in patients, especially individuals with moderate to high risk of stroke incidence [26]. Standard recommendations for the care of individuals with AF include the administration of warfarin to reduce the risk of stroke. If these recommendations were put into clinical practice, tens of thousands of strokes may be avoided [27]. Anticoagulation therapy also significantly reduces the occurrence of mortality among AF patients with a risk of thromboembolic events, according to the CHA_2_-DS_2 _scoring system.

Direct oral anticoagulants (DOACs) are currently routinely used to lower this risk as a consequence of the findings of several randomized controlled studies (RCTs) [28,29]. They have been shown to have better risk-benefit profiles than warfarin, with notable decreases in stroke, cerebral hemorrhage, and death [30]. Warfarin's safety and effectiveness have been recognized for decades, but less is known about the DOACs' integration into standard clinical practice. Due to their shorter half-lives and potential for decreased efficacy with poor adherence, DOACs continue to raise concerns. Due to the lack of laboratory monitoring to determine therapeutic levels, it is crucial to determine how patient characteristics and the various DOACs currently on the market affect adherence to DOACs [31].

There is an increased risk of systemic embolism and ischemic stroke with nonvalvular AF. When compared to a placebo, warfarin, an adjusted-dose prophylactic, prevents stroke by 62%. However, a seven- to tenfold increase in intracranial hemorrhage, especially in senior individuals, worsens their condition and increases the likelihood of a shorter lifespan [32]. Vitamin K antagonists interact with several substances, including food and medications, necessitating dose modifications and ongoing observation [33]. The undertreatment of individuals at high risk for stroke is a result of its unpredictable nature. An oral direct thrombin inhibitor called ximelagatran is being studied as a thromboembolism preventative and therapeutic anticoagulant [34].

Ximelagatran, a medication known as an oral direct thrombin inhibitor, and warfarin were compared for their ability to prevent strokes and embolisms in the system in a double-blinded, randomized, multicenter experiment. About 3922 participants enrolled with nonvalvular AF and other stroke risk variables participated in the experiment. All strokes and systemic embolic events were the main endpoint. About 88 patients had primary events throughout the course of 6405 patient-years of follow-up. For 68% of the treatment duration, the mean international normalized ratio (INR) with warfarin was within the desired range. Warfarin and ximelagatran had main event rates of 1.2% and 1.6%, respectively, per year. The difference in rate was 0.10% each year when all-cause mortality was considered. Major bleeding rates were similar between treatment groups; however, ximelagatran had decreased overall hemorrhage. In 6.0% of individuals receiving ximelagatran, serum alanine aminotransferase levels increased to more than threefold the maximum value of normal. The findings demonstrate the superiority of fixed-dose oral ximelagatran over properly managed warfarin in avoiding thromboembolism in individuals with AF who need long-term anticoagulant treatment [35].

## Review

Methodology

We included cohort and observational studies along with RCTs due to the distinctive features of the study. The Medical Subject Headings (MeSH) terms and keywords utilized for the search were anticoagulants (PCOS), vitamin K antagonists, anticoagulation therapy, treatment outcome OR therapeutics, NOACs, atrial fibrillation, stroke, warfarin, and stroke risks among numerous other research terms that were employed in an electronic search on Elsevier, PubMed Online, NIH, Wiley, EMBASE, EBSCO, and Scopus along with Google Scholar. The literature-based systemic review for the most recent research employed the Preferred Reporting Items for Systematic Reviews and Meta-Analyses (PRISMA) strategy. It was thoroughly reviewed and analyzed to determine if every component met the criteria for inclusion. It was evaluated and examined. If they satisfied all inclusion criteria, they were included in the research.

Inclusion criteria

The inclusion criteria for this study encompass research involving adult patients diagnosed with AF. These studies should primarily concentrate on evaluating the efficacy of anticoagulant treatments, particularly in terms of stroke risk reduction as a measurable outcome. Additionally, the selected studies must involve various modalities of anticoagulant treatment. Furthermore, the requirement dictates that the studies must be published in peer-reviewed journals, ensuring a level of quality and reliability in the research findings.

Exclusion criteria

The exclusion criteria for this study entail excluding studies with participants who do not meet the criteria for anticoagulant therapy in patients with AF. Additionally, studies that lack a specific focus on investigating anticoagulant interventions are excluded. Furthermore, studies with inadequate sample sizes or poor study design, such as case reports, are excluded from consideration. Non-English language studies are excluded unless they are deemed essential for inclusion. Moreover, studies with incomplete or unavailable data for the outcomes of interest and those lacking relevant outcome data related to stroke risk reduction have also been excluded from the analysis.

Table 1 presents a bias assessment of research studies using the Cochrane risk assessment tool. Each study is evaluated based on criteria such as randomization process, deviation from intervention, missing outcome data, outcome measurement, selection of reported result, and overall bias risk. The table categorizes each study's risk of bias as low, some concerns, or low risk overall.

**Table 1:** Bias assessment of research studies via the Cochrane risk assessment tool Overall bias risk: Summary assessment of the overall risk of bias in the study, categorized as "low," "some concerns," or "low risk"

Study	Randomization process	Deviation from intervention	Missing outcome data	Measurement of outcome	Selection of reported result	Overall, bias risk
	D1	D2	D3	D4	D5	Results
Yao et al. (2016) [36]	Low	Low	Low	Low	Low	Low risk
Brass et al. (1997) [37]	Low	Low	Low	Low	Low	Low risk
Borne et al. (2017) [38]	Low	Low	Low	Low	Low	Low risk
Wolf (1991) [2]	Low	Low	Low	Low	Low	Low risk
Samsa (2000) [39]	Low	Low	Low	Low	Some concerns	Some concerns
Olsson et al. (2003) [40]	Low	Low	Low	Low	Low	Low risk
Albers et al. (2006) [41]	Low	Low	Low	Low	Low	Low risk
Johnson et al. (2005) [42]	Low	Low	Low	Low	Low	Low risk
Jacobs (2009) [43]	Low	Low	Low	Some concerns	Some concerns	Some concerns

Results and discussion

**Figure 1:**
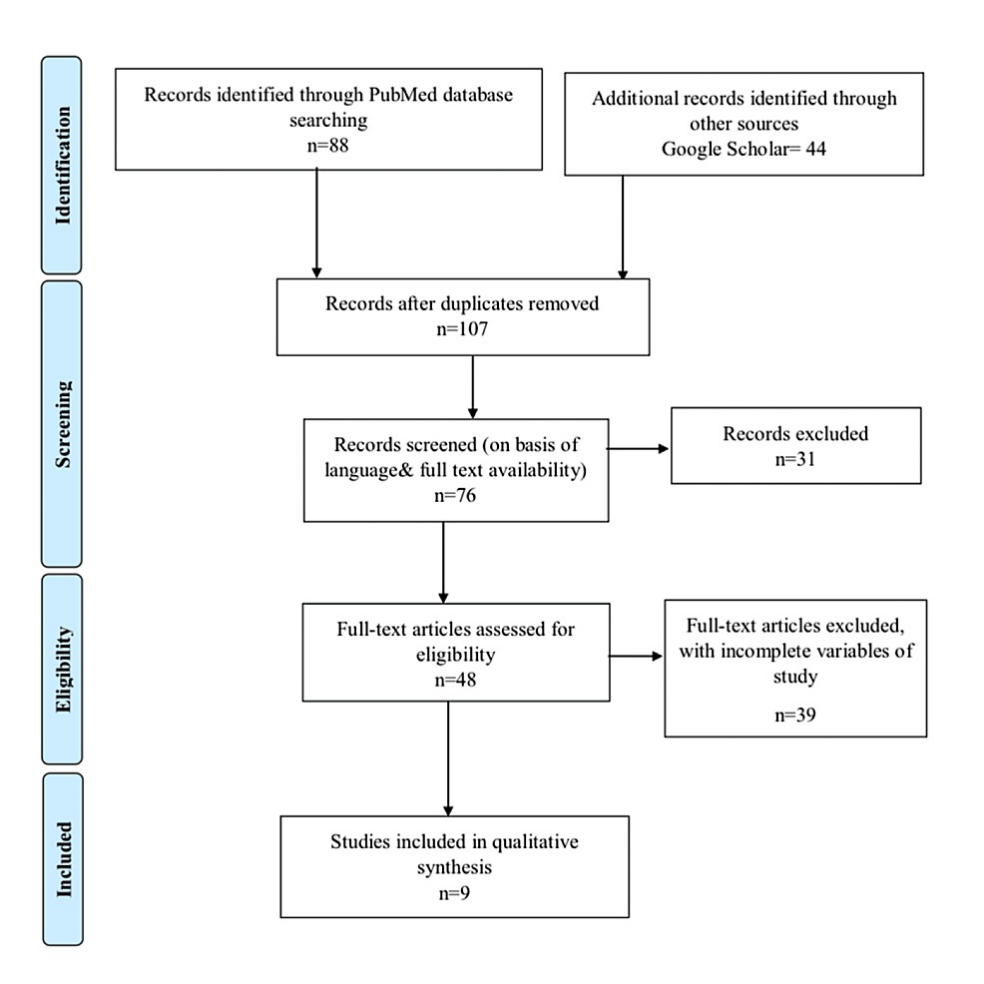
The PRISMA figure PRISMA: Preferred Reporting Items for Systematic Reviews and Meta-Analyses

We identified 132 research papers from electronic searches of databases, i.e., Scopus, EBSCO, Elsevier, Springer, PubMed, and BMC. We started screening studies by removing the duplicate research, which left us with 107 articles. We further filtered the articles on the absence of desired components in research and the availability of the full text of articles. After the screening process, we were left with 48 researches which were narrowed down to nine after a thorough research study. The total number of participants, mean age, and other parameters were noted (Figure 1).

Table 2 summarizes several studies investigating the management and outcomes of AF patients, along with their characteristics and results.

**Table 2:** Characteristics of studies CHA2DS2-VASc: risk based on the presence of congestive heart failure, hypertension age 65–74 years, age ≥75 years, diabetes mellitus, prior stroke or transient ischemic attack, vascular disease, and sex category; NOACs: non-vitamin K antagonist oral anticoagulants; HTN: hypertension; CAD: coronary artery disease; DM: diabetes mellitus; PAD: peripheral artery disease; DOAC: direct oral anticoagulants; PDC: proportion of days covered; TTR: time-in-therapeutic range

Study	Year	Location	Type of study	Diagnosis	N	Age	Gender	Anticoagulant	Time period	Comorbidities	Outcome
Yao et al. [36]	2016	USA	Retrospective cohort study	Atrial fibrillation	64661	73	M = 36339 F = 28322	Warfarin (59.1%), dabigatran (15.8%), rivaroxaban (19.1%), apixaban (6.0%)	1.1 year follow-up	DM, congestive heart failure, HTN, liver disease, major bleeding event	Periods without anticoagulation medication did not contribute to stroke among individuals who had CHA_2_DS_2_-VASc scores of 0 or 1. However, stopping anticoagulation for less than three months was linked to a substantial decrease in bleeding. NOACs may somewhat alleviate the substandard administration of anticoagulant adherence
Brass et al. [37]	1997	USA	Observational cohort study	Atrial fibrillation	488	85	M= 180 F= 308	Warfarin (38%)	6 months	Dementia, metastatic cancer, prior bleeding event, HTN, DM, congestive heart failure, CAD	A good potential for senior preventive measures for stroke is represented by more significant warfarin usage among those with atrial fibrillation
Borne et al. [38]	2017	USA	Retrospective cohort study	Nonvalvular atrial fibrillation	2882	67.4	M=2792 F=90	Dabigatran (72.7%), rivaroxaban (19.8%), apixaban (7.50%)	5 years	Congestive heart failure, DM, HTN, PAD	The percentage of participants who were nonadherent, PDC 0.80, was 27.6% overall and differed by DOAC. Elevated risk of death and stroke was linked to decreased dabigatran compliance (HR 1.07; 1.03-1.12 per 0.10 reduction in PDC). Decreased adherence has been correlated to an increased risk of death and having a stroke
Wolf [2]	1991	USA	Longitudinal study	Atrial fibrillation, cardiac failure, CAD, CHD	5209	50		warfarin	34 years follow-up	HTN, CAD, cardiac failure	Atrial fibrillation was the sole cardiovascular condition to exert an independent effect on stroke incidence. The strong autonomous impact of atrial fibrillation described here is consistent with the results in which warfarin anticoagulation mitigated >50% of stroke occurrences
Samsa [39]	2000	USA	Retrospective study	Atrial fibrillation	660	68.8	M = 344 F = 316	Warfarin (34.7%), others		Anemia, active bleeding, smoking, DM, HTN, cerebral aneurysm, CVD	It was found that despite the relation of warfarin to decreased stroke occurrences, it was not prescribed to patients even though they were eligible for the anticoagulant. This could lead to decreased stroke episodes among atrial fibrillation patients
Olsson et al. [40]	2003	Europe, Asia, Australia	Randomized control trial	Nonvalvular atrial fibrillation	1740	70.3	M = 1158 F = 582	Ximelagatran	17.4 months	HTN, DM, stroke, embolism	By intention to treat, all-cause mortality was 79 patients in the warfarin-assigned group (3·2% per year) and 78 patients in the ximelagatran group (3·2% per year). About 33 vascular deaths occurred in patients assigned to warfarin and 40 in those on ximelagatran (p = 0·478). Nine fatal strokes happened in the warfarin group and ten in the ximelagatran group. Fixed-dose oral ximelagatran was at least as efficacious as properly managed warfarin in vulnerable individuals with atrial fibrillation for preventing stroke and embolism of the system
					1703	70.1	M = 1156 F = 547	Warfarin			
Albers et al. [41]	2005	USA, Canada	Double-blind, randomized, multicenter trial	Nonvalvular atrial fibrillation	1960	71.6	M = 1365; F = 595	Ximelagatran	20 months	HTN, DM, embolism	Warfarin and ximelagatran had main event rates of 1.2% and 1.6%, respectively, on an annual basis (absolute difference, 0.45% per year; 95% confidence interval, 0.13% to 1.03% per year; P.001). The direct thrombin inhibitor ximelagatran was not superior to well-controlled warfarin within the prespecified margin of 2.0% per year to prevent stroke and systemic embolism in this trial, enrolling relatively high-risk patients with nonvalvular atrial fibrillation
					1962	71.6	M = 1353; F = 609	Warfarin			
Johnson et al. [42]	2005	Australia	Retrospective observational cohort study	Atrial fibrillation	228	81.1	M = 95; F = 133	Warfarin (53.3%)	28 months		In this investigation, 17 strokes with an annual occurrence rate of 2.6% happened in individuals elderly 76 and older after initiating warfarin medication. Formerly, it was shown that atrial fibrillation put 80% of hospitalized patients at elevated risk for stroke
Jacobs [43]	2009	UK	Retrospective observational cohort study	Atrial fibrillation, dementia	106		M = 80; F = 26	Warfarin (85%), acetylsalicylic acid ASA (15%)	12 months	DM, ischemic heart attack	About 17% of patients had the low score of CHADS2 score, whereas 81 patients had a score of 2-4 and 7% with high score. Although having a relatively modest TTR, a substantial majority of patients (85%) who had chronic AF and also had falls or dementia were taking warfarin, which had low 12-month risks of stroke, bleeding, and mortality. The death rate for patients who had memory impairment and/or falls was substantial (about 45%)

In earlier research on oral anticoagulants, individuals with comparable stroke risk at baseline but varying periods of anticoagulation discontinuation were frequently compared. With other potential confounders taken into account, this study aimed to evaluate individuals at similar risk but different levels of anticoagulant discontinuation. Significant bleeding may need treatment discontinuation and raise the risk of stroke [44]. Poor anticoagulant medication compliance is less understood now that improving physician compliance with prescription recommendations has become a priority. However, many individuals have trouble adhering to warfarin over the long term [45]. Moreover, the timing of anticoagulation initiation in patients with AF has emerged as a critical consideration. A recent meta-analysis comparing early (≤7 days) vs delayed (>7 days) initiation of anticoagulation after acute stroke in patients with AF found no significant differences in the risk of recurrent ischemic stroke, intracranial hemorrhage, or mortality between the two groups [46].

In a district hospital context, the research sought to assess computer-assisted oral anticoagulation's long-term effectiveness and hazards for nonrheumatic AF (NRAF). It is worth considering the role of anticoagulation in subclinical AF, where individuals may not exhibit overt symptoms but still face an increased risk of stroke and other thromboembolic events. Recent studies have highlighted the importance of anticoagulation therapy in this subpopulation, emphasizing the need for early detection and intervention to prevent adverse outcomes [47]. With computer-assisted anticoagulation, 38.3% of patients were uncontrolled during 1484 patient years. Age had no significant impact on INR management, although it substantially impacted testing regularity. The annual risks of thrombosis, stroke throughout the year, and bleeding complications were 0.76%, 0.35%, and 0.84%, respectively. At the time of their clinical occurrence, patients with thromboembolic complications and hemorrhagic sequelae were significantly more likely to be experiencing under-anticoagulation and over-anticoagulation. The study came to the conclusion that computerized long-term oral anticoagulation for NRAF is effective and secure, with anticoagulation management, bleeding events, thromboembolic instances, and incidences of stroke equivalent to those reported in significant clinical trials [47].

In a survey of doctors conducted across the country by McCrory et al., it was almost universally agreed that anticoagulant therapy was successful [48]. In a different poll, over 95% of doctors suggested treatment to prevent thromboembolic consequences; among patients with AF, warfarin medication was favored by over 80% of doctors, while virtually all others advised antiplatelet therapy [48,49].

Periods without anticoagulation medication did not contribute to stroke among individuals who had CHA_2_DS_2_-VASc scores of 0 or 1. However, stopping anticoagulation for less than three months was linked to a substantial decrease in bleeding. NOACs may somewhat alleviate the substandard administration of anticoagulant adherence. In individuals with a CHA_2_DS_2_-VASc score of 2, compliance with treatment [D5] seems to be of the utmost importance. Still, in individuals with an overall rating of 0 or 1, the advantages of anticoagulation are unlikely to outweigh the disadvantages [36].

Furthermore, studies conducted in developing countries like India have highlighted the challenges associated with poor medication compliance among patients with nonvalvular AF. A research by Sharma et al. (2020) found suboptimal anticoagulation outcomes in a tertiary care center in North India, indicating gaps in adherence to treatment protocols [5]. Similarly, a survey by Joshua and Kakkar (2015) revealed significant lacunae in patient knowledge about oral anticoagulant treatment, suggesting potential barriers to effective medication management [50].

A negative relationship between anticoagulant level and stroke was reported. With its ability to cleave fibrinogen into fibrin and activate platelets and several coagulation factors, thrombin plays a crucial part in the process of thrombogenesis. Even while thrombin is eventually inhibited by other anticoagulant medications like warfarin and heparin, most of them do so by indirect processes [51]. Melagatran, a direct inhibitor of soluble and fibrin-bound thrombin, is bioconverted from oral ximelagatran. Ximelagatran's anticoagulant effectiveness is superior to warfarin and dalteparin for the prevention and treatment of venous thromboembolism [52].

In research, it was shown that 80% of hospitalized patients with AF were at high risk for stroke since they had an age over 75 and had not less than a single additional risk factor for stroke, leading to the observation that 17 strokes took place in patients aged 76 and older after initiating warfarin medication, with an average yearly rate of 2.6% [53]. Similar rates in this study are anticipated to exist. In individuals over 75 with one or more risk factors, the collected information and Stroke Prevention in Atrial Fibrillation (SPAF) II indicated an annual incident prevalence of thromboembolism of 1.2%3 and 4.2%12, respectively. According to the Kaiser Permanente Medical Care Program, the risk of stroke caused by ischemic attack was 1.11 per 100 patient-years for patients using warfarin and 1.88 for those who weren't [54-56]. Only five (29%) of the strokes in this research happened, while the patient's INR was therapeutic (between two and three), and six (35%) occurred when warfarin was not being taken either for a short or long time by the patient. In the group of individuals given warfarin in the pooled data, 30% of stroke victims and 43% of older who had thromboembolic incidents within the group given warfarin in SPAF II weren't taking it at the time. This illustrates the dangers of an INR below therapeutic levels. When feasible, a different anticoagulant that can be quickly started and stopped, such as heparin, should be considered [42].

Clinical studies are the primary source of information about the risk of thromboembolic stroke and the effectiveness of warfarin in the elderly. When correlated to the 41% of senior patients overall who had chronic AF, another study of at-risk older persons with AF had a higher prescription rate for warfarin at 85% [43]. The Birmingham AF Treatment of the Aged (BAFTA) study found that warfarin and ASA significantly reduced stroke risks in geriatric patients with AF aged ≥75 years. The study reported annual stroke risks of 1.8% and 3.8%, respectively. However, the reported stroke rate (2%) was lower than the predicted rate of 4.8%. The study also found that 6% of patients treated with warfarin experienced major hemorrhage, which was lower than the BAFTA trial's 1.4%. This discrepancy may be because the INR was elevated above the therapeutic range in only 4% of cases [57].

In completely blind research, 336 individuals suffering from chronic nonrheumatic AF got aspirin 75 mg once a day and 336 placebos, whereas 335 participants administered warfarin anticoagulation openly. Every patient was monitored for two years. A thromboembolic complication (stroke, acute ischemic attack, or embolic consequences to the viscera and extremities) was the main outcome. Mortality was the extraneous outcome. In contrast to the aspirin and placebo groups, which weren't shown to vary statistically, the rate of complications associated with thromboembolism and vascular fatalities was significantly decreased in the warfarin cohort. Compared to 20 individuals taking aspirin and 21 receiving a placebo, five patients taking warfarin experienced thromboembolic events. Compared to two patients on aspirin and none on a placebo, 21 individuals on warfarin had to be discontinued due to nonfatal bleeding problems. So, in individuals with persistent nonrheumatic AF, anticoagulation treatment with warfarin may be advised to avoid thromboembolic consequences [58].

There is a lot of data to suggest that stroke patients with AF have a poorer prognosis than those without AF, including increased death, degree of severity, and frequency of recurrence as well as more severe impairments in functioning and reliance. According to estimates, the long-term costs of stroke ranged from US $24 991 for a moderate stroke over five years to US $142 251 for a large ischemic stroke over a lifetime (prices from 2004). A major ischemic stroke can often cost three times as much as a minor stroke. However, cost-effectiveness models for anticoagulation in patients with AF have relied on average (rather than AF-specific) statistics on the cost of stroke, and the majority have utilized the severity of stroke proportions generated from research studies, which may be different from those in real practice. Since they neglect to account for the worse outcomes linked to cardioembolic strokes, current fiscal projections underestimate the cost advantages of anticoagulation for avoiding the occurrence of strokes. The prescription of anticoagulants to patients can reduce the incidence of stroke as well as decrease the economic burden on individuals and healthcare forces and utilities, indicating the dire need for anticoagulation therapies among patients of AF to be considered seriously [59].

This study had a lot of potential for extensive research in this domain. Some limitations of this review could be improved for better outcomes and a better depiction of anticoagulant utilization for AF patients. Rates of stroke annually with mortality associated with stroke, as well as survival analysis of AF patients, should also be considered in depth. Comparative studies of various anticoagulants and the effectiveness of comparisons of anticoagulants to antiplatelets should also be included. Side effects experienced by patients due to consumption of anticoagulants, cause of nonadherence to anticoagulants by patients as well as lack of recommendation of anticoagulants by healthcare workers should also be investigated along with short- and long-term side effects of anticoagulants among AF patients.

## Conclusions

The review studied the role of anticoagulation in preventing stroke among nonvalvular AF. It was found that various anticoagulants, such as warfarin, ximelagatran among many more, are prescribed to patients who have the potential to reduce the incidence of stroke as well as alleviate their likelihood of developing other thromboembolic events that can decrease their quality of life. Economic and psychological burdens associated with diminished functionality can be prevented by anticoagulant therapy among AF patients, therefore reducing their economic and social burden. This is due to the negative association between stroke among AF patients and anticoagulation consumption.
